# C9orf72 deficiency promotes motor deficits of a C9ALS/FTD mouse model in a dose-dependent manner

**DOI:** 10.1186/s40478-019-0685-7

**Published:** 2019-03-04

**Authors:** Qiang Shao, Chen Liang, Qing Chang, Wei Zhang, Mei Yang, Jian-Fu Chen

**Affiliations:** Center for Craniofacial Molecular Biology, University of Southern California (USC), Los Angeles, CA 90033 USA

**Keywords:** C9orf72, Motor deficits, C9ALS/FTD, Mice

G4C2 hexanucleotide repeat expansions in the first intron of *C9ORF72* are the most common cause of familial amyotrophic lateral sclerosis (ALS) and frontotemporal dementia (FTD) (collectively, C9ALS/FTD) [4, 6, 11, 14]. Haploinsufficiency (loss-of-function) of C9ORF72 protein is a key proposed disease mechanism which may act in parallel with gain-of-function mechanisms, including toxic RNAs from repeat transcription and dipeptide repeat proteins (DPRs) from repeat-associated non-AUG (RAN) translation [5, 9, 17]. However, the effect of *C9orf72* deficiency in the background of gain-of-function has not been examined in vivo. Neither heterozygous nor homozygous knockout (KO) of *C9orf72* in neurons leads to motor deficits in mice [8]. Recently, gain-of-function mouse models were generated using a *C9ORF72* bacterial artificial chromosome (BAC) from C9ALS/FTD patient DNA under the control of the endogenous regulatory elements. Interestingly, three out of four of these *C9-BAC* transgenic mice did not develop motor behavior deficits, even at advanced ages [7, 12, 13]. Since these *C9-BAC* mouse models contain elevated C9orf72 proteins from the endogenous mouse gene, we hypothesized that C9orf72 provides neuroprotective effects against motor deficits in *C9-BAC* mice.

To test this hypothesis and investigate the in vivo significance of C9orf72 haploinsufficiency, we crossed *C9orf72*^*+/−*^ mice with *C9-BAC* mice and examined the consequences of C9orf72 protein dose reduction (loss-of-function) in the background of *C9-BAC* (gain-of-function). We found that *C9orf72* loss and haploinsufficiency exacerbate motor behavior deficits in a dose-dependent manner, and this occurs early in the course of pathogenesis (4 months of age). Among the four published *C9-BAC* mouse models, we selected the one with motor deficits (we refer to this *C9orf72 BAC*^*Tg/+*^ model as the *C9-BAC* line here) [10]. To reduce C9orf72 protein levels at different doses, we crossed *C9orf72*^*+/−*^ and *C9-BAC* mice for two generations. We isolated proteins from brain tissues and confirmed the expected C9orf72 protein dose reduction (Fig. 1a, Additional file 1: Figure S1A). The unchanged protein level of Atg101, which is associated with the C9orf72/Smcr8 complex based on our previous study [16], suggests the specificity of C9orf72 reduction (Fig. 1a, Additional file 1: Figure S1A).

**Fig. 1:**
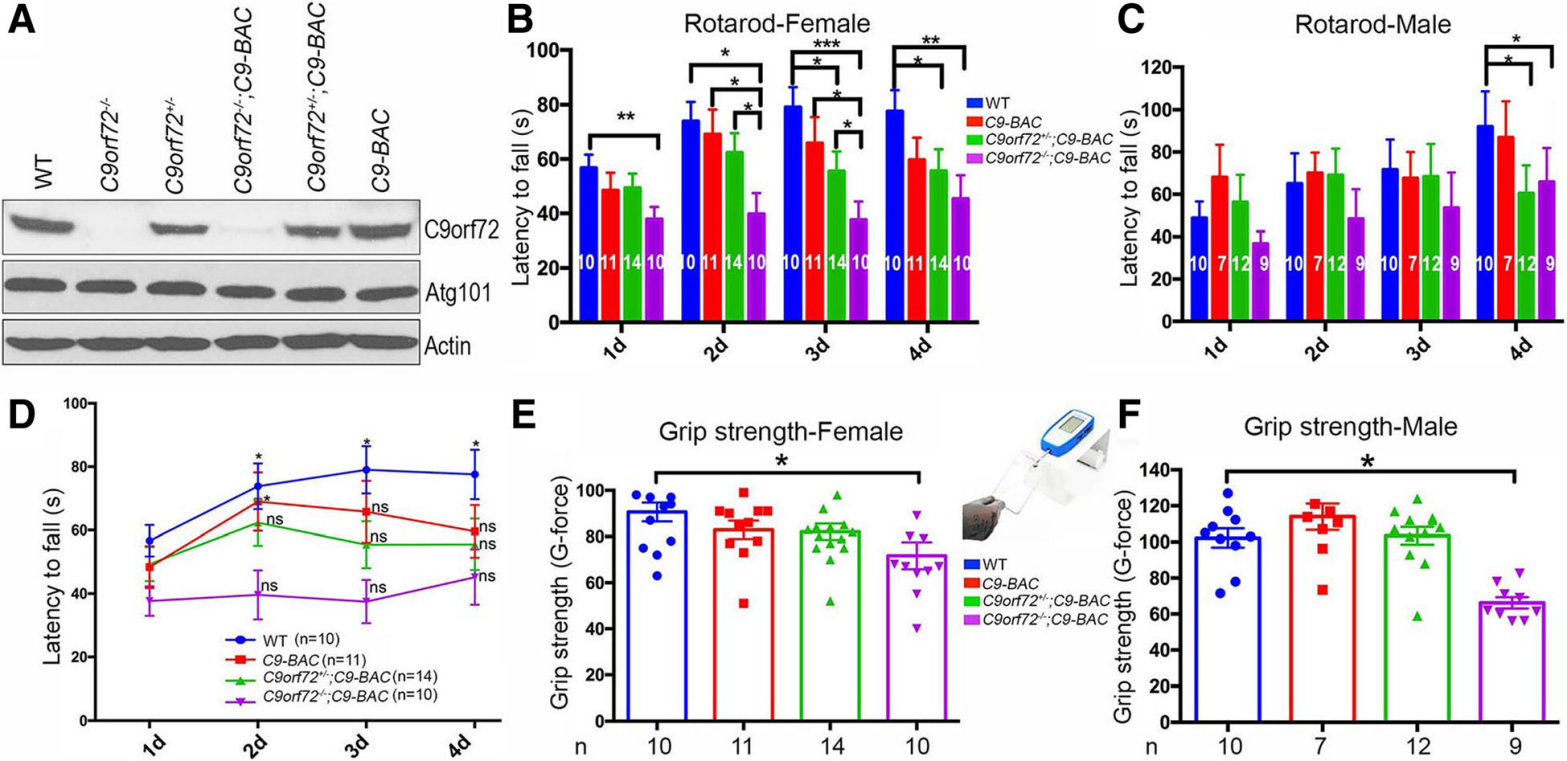
*C9orf72* dose is critical for motor deficits in C9ALS/FTD mouse models. **a** Western blot analysis of C9orf72 and Atg101 protein levels in 2-month-old mouse cortex. β-Actin serves as the loading control. **b**, **c** Accelerating rotarod test was performed on 4-month-old mice to examine the latency to fall of females (**b**) and males (**c**). *C9orf72* deficiency decreases the latency to fall of *C9-BAC* female mice in a dose-dependent manner. **d** A 4-consecutive-day rotarod assay reveals defective motor learning in *C9orf72*^*+/−*^*;C9-BAC* and *C9orf72*^*−/−*^*;C9-BAC* female mice in comparison to WT. **e**, **f** Grip strength test was performed to measure front paw strength in 4-month-old females (**e**) and males (**f**). All data are presented as mean ± SEM using numbers (n) of mice as indicated. Statistical analyses were performed with one-way ANOVA with Bonferroni’s post hoc test (*p < 0.05, **p < 0.01, ***p < 0.001, N.s represents no significant difference detected in measurement of 2d, 3d, or 4d in comparison to that of 1d)

To study effects of *C9orf72* deficiency on the motor behaviors of *C9-BAC* mice, we monitored a cohort of mice [20 WT (10 females + 10 males), 18 *C9-BAC* (11 females + 7 males), 26 *C9orf72*^*+/−*^;*C9-BAC* (14 females + 12 males), and 19 *C9orf72*^*−/−*^*;C9-BAC* (10 females + 9 males)]. We excluded *C9orf72*^*+/−*^ and *C9orf72*^*−/−*^ mice for the following reasons: *C9orf72* heterozygous and homozygous KO mice exhibited no neurodegeneration and motor deficits based on previous studies [8]; complete deletion of *C9orf72*, which does not occur in C9ALS/FTD patients, led to autoimmune disorders and reduced survival in mice [1], which may complicate large-scale behavior and survival studies. We found that there were no significant differences among the four tested groups in their survival around 4 months, when behaviors were assessed. They also exhibited similar body weights, taking the sex of the mice into account (Additional file 1: Figure S1B-1C). To examine their general anxiety levels, we performed an open field test [3]. *C9-BAC* mice with different C9orf72 levels behaved similarly in total distance traveled, distance traveled in the center, and time spent in the center (Additional file 1: Figure S1E-1G).

We next examined their motor coordination and balance using an accelerating (4–40 rpm in 5 min) rotarod test. Mice were given five trials per day, with an inter-trial interval of 20 min, for 4 consecutive days. A C9orf72 dose-dependent decrease in latency to fall was detected in *C9-BAC* female mice (Fig. 1b), and in *C9-BAC* male mice on day 4 of the rotarod assay (Fig. 1c). These results suggest that motor coordination is sensitive to C9orf72 protein levels in *C9-BAC* mice. We further analyzed motor learning in female mice. WT mice exhibited an increase in latency to fall over the course of 4 consecutive days, indicating active motor learning (Fig. 1d). Latency to fall of *C9-BAC* mice was increased on day 2 but dropped on days 3 and 4 (Fig. 1d). Importantly, there was no increase in latency to fall from day 1 to day 4 in *C9orf72*^*+/−*^*;C9-BAC* and *C9orf72*^*−/−*^*;C9-BAC* animals (Fig. 1d). These results suggest that *C9orf72* deficiency impaired motor coordination and motor learning of *C9-BAC* mice in a dose-dependent manner.

To examine motor strength, we measured forearm grip strength and found that it was significantly reduced in both male and female *C9orf72*^*−/−*^*;C9BAC* animals compared to other genotypes (Fig. 1e, f). Lastly, we measured the maximal speed at which each animal fell from the rotarod device. Results showed that *C9orf72* deficiency, in a dose-dependent manner, decreased the maximum speed at which *C9-BAC* mice fell (Additional file 1: Figure S1H, S1I), which is consistent with the data on their latency to fall.

The rotarod assay revealed more evident motor impairment in female mice than in male mice. This could be due to toxic gain-of-function since *C9-BAC* female mice exhibited earlier and more pronounced abnormalities than male mice [10]. It will be important to examine using similar cohorts of mice whether motor neurons (MNs) degenerate or reduce in number in a C9orf72 dose-dependent manner and whether these deficits correlate with the observed motor behavior deficits. Future studies should also investigate whether C9orf72 exhibits dose-dependent effects in the three other *C9-BAC* mouse models [7, 12, 13]. It will be informative to examine the effects of *C9orf72* deficiency in the background of adeno-associated virus (AAV)-mediated G4C2 repeat expression [2]. Our study indicates that C9orf72 haploinsufficiency contributes to disease onset in a mouse model by exacerbating the pathogenic effects of RNA/DPR-mediated neurotoxicity. Together with a recent report on patient iPSC-derived MNs [15], this study suggests indeed that we should focus more on the combination of loss- and toxic gain-of-function. Together, for the first time, our mouse genetic studies showed that *C9orf72* loss or haploinsufficiency in a gain-of-function mouse model of C9ALS/FTD exacerbate motor behavior deficits in a dose-dependent manner, demonstrating the importance of C9orf72 haploinsufficiency in vivo.

## Additional file

Additional file 1: **Figure S1.** Characterization of *C9-BAC* mice with C9orf72 dose reduction. (A) Quantification of C9orf72/Atg101 protein levels. Data are presented as mean ± SEM from three independent experiments. (B, C) Body weight of female (B) and male (C) mice at 4 months of age. (D-G) Open field test was performed on 4-month-old mice to examine the total distance traveled (E), distance traveled in the center (F), and percentage of time spent in the center (G). (H, I) Quantification of the maximal g force from five trials of rotarod assay. *C9orf72* deficiency decreases the maximal g force of *C9-BAC* female mice in a dose-dependent manner. All data are presented as mean ± SEM using numbers (n) of mice as indicated. Statistical analyses were performed with one-way ANOVA with Bonferroni’s post hoc test (*p < 0.05, **p < 0.01, ***p < 0.001, n.s represents no significant difference detected). (PDF 2855 kb)
