# Supplementary figures and images for: C9orf72 deficiency promotes motor deficits of a C9ALS/FTD mouse model in a dose-dependent manner
Source: Acta Neuropathol Commun. 2019 Mar 4;7:32. doi: 10.1186/s40478-019-0685-7 (PMC6398253; doi:10.1186/s40478-019-0685-7)

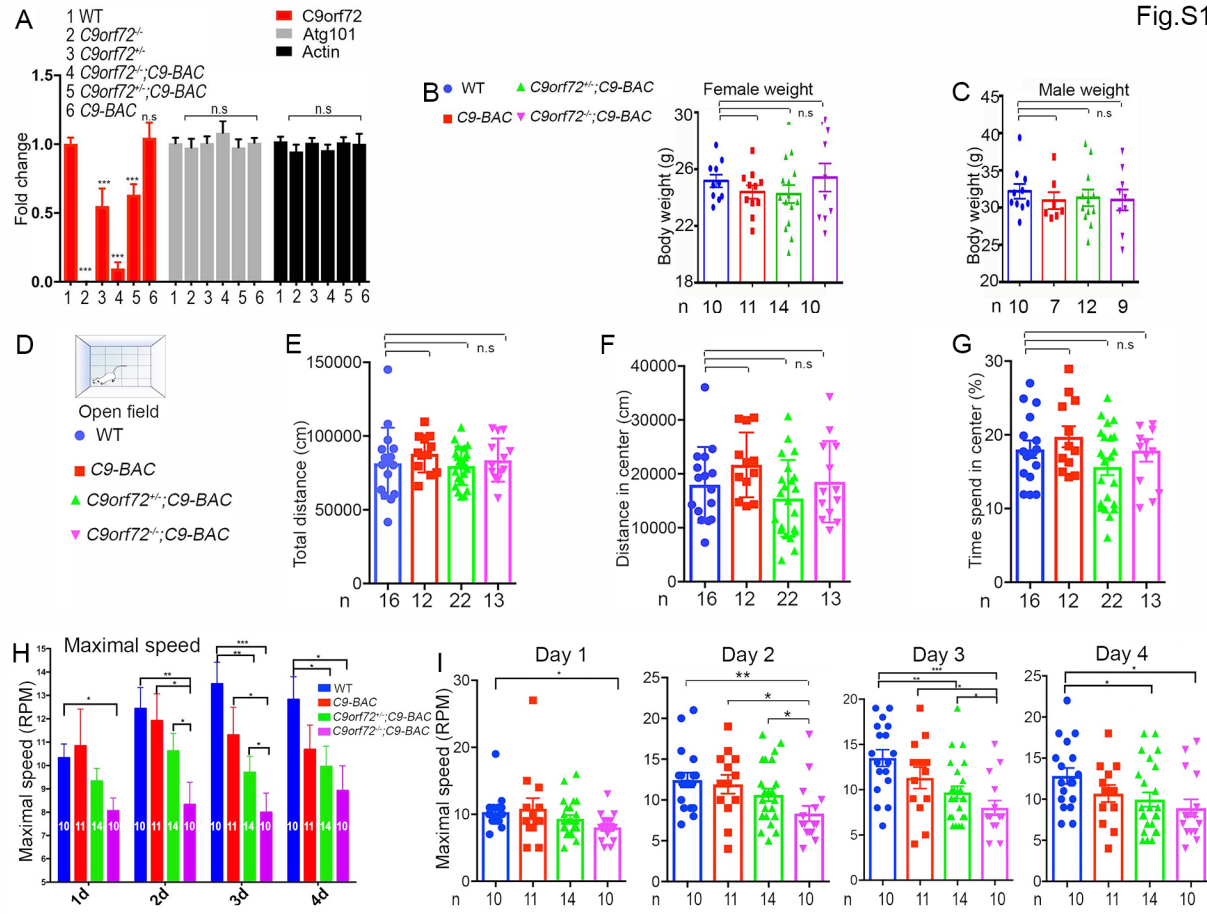

Supplement: Supplementary file 1 — Figure S1. Characterization of C9-BAC mice with C9orf72 dose reduction. (A) Quantification of C9orf72/Atg101 protein levels. Data are presented as mean ± SEM from three independent experiments. (B, C) Body weight of female (B) and male (C) mice at 4 months of age. (D-G) Open field test was performed on 4-month-old mice to examine the total distance traveled (E), distance traveled in the center (F), and percentage of time spent in the center (G). (H, I) Quantification of the maximal g force from five trials of rotarod assay. C9orf72 deficiency decreases the maximal g force of C9-BAC female mice in a dose-dependent manner. All data are presented as mean ± SEM using numbers (n) of mice as indicated. Statistical analyses were performed with one-way ANOVA with Bonferroni’s post hoc test (*p < 0.05, **p < 0.01, ***p < 0.001, n.s represents no significant difference detected). (PDF 2855 kb) [file 40478_2019_685_MOESM1_ESM.pdf]
